# Does Intolerance of Uncertainty Influence Social Anxiety Through Rumination? A Mediation Model in Emerging Adults

**DOI:** 10.3390/bs15050687

**Published:** 2025-05-16

**Authors:** Kıvanç Uzun, Süleyman Ünlü, Gökmen Arslan

**Affiliations:** 1 Department of Psychological Counselling and Guidance, Faculty of Education, Uşak University, Uşak 64200, Türkiye; 2 Department of Turkish Education, Faculty of Education, Uşak University, Uşak 64200, Türkiye; 3 Department of Psychological Counselling and Guidance, Faculty of Education, Burdur Mehmet Akif Ersoy University, Burdur 15030, Türkiye

**Keywords:** emerging adults, social anxiety, intolerance of uncertainty, rumination, mediation model, cognitive vulnerabilities, collectivist cultures, psychological well-being, REBT, CBT

## Abstract

In order to support the psychological well-being of individuals in emerging adulthood, it is important to understand the cognitive mechanisms that may contribute to social anxiety. In particular, intolerance of uncertainty is considered an important factor associated with anxiety levels among emerging adults. Accordingly, this cross-sectional study investigated whether intolerance of uncertainty is associated with social anxiety through ruminative thoughts. The study group consisted of 405 individuals living in Türkiye and in emerging adulthood, 70.60% (*n* = 286) of whom were female and 29.40% (*n* = 119) of whom were male. The ages of the participants selected by convenience sampling method ranged between 18 and 25 years, with a mean age of 21.73 years (*SD* = 1.87). In this study, data were collected using a demographic information form and three different self-report scales. The findings revealed that intolerance of uncertainty was significantly associated with higher levels of social anxiety (*β* = 0.22, *p* < 0.001). Moreover, rumination emerged as a significant cognitive mediator in this relationship (*β* = 0.23, *p* < 0.001). The indirect effect was statistically significant, indicating that individuals who experience high intolerance of uncertainty may be more prone to social anxiety when they also engage in ruminative thinking. Together, intolerance of uncertainty and rumination explained 26% of the variance in social anxiety (*R*^2^ = 0.26). These results emphasize the importance of targeting both intolerance of uncertainty and rumination in interventions aimed at reducing social anxiety among emerging adults.

## 1. Introduction

Emerging adulthood is a developmental period covering the transition period between adolescence and adulthood, in which individuals between the ages of approximately 18–25 years are involved, and, in this period, processes such as identity formation, gaining independence, and making future plans are intensely experienced (Arnett, 2000, 2007). This period also increases the risk of experiencing social anxiety due to increased social relationships and efforts to exist in work or educational environments (LeBlanc et al., 2020). When social anxiety is not adequately addressed during this developmental period, it can have negative effects on individuals’ academic achievement, performance at work, and general social functioning (Özer & İlhan, 2020; Vilaplana-Pérez et al., 2021). This may lead to a loss of self-confidence by damaging the individual’s self-knowledge process, a decrease in productivity, and thus a decrease in the potential to participate productively in society (LeBlanc et al., 2020; Potterton et al., 2022). In Türkiye, the one-year and lifetime prevalence of social phobia among university students have been reported to be 20.90% and 21.70%, respectively, indicating that social anxiety is a highly common concern in this population (Gültekin & Dereboy, 2011). In this context, social anxiety, especially in emerging adulthood, emerges as a critical variable that should be emphasized in order to support the healthy development of individuals.

Social anxiety can be defined as a state of intense worry and discomfort that individuals feel due to the fear of being evaluated or criticized by others in social settings (Hyett & McEvoy, 2018; Morrison & Heimberg, 2013). This condition is triggered by individuals feeling inadequate or vulnerable to negative evaluation in social interactions and can reduce quality of life by limiting social functioning over time (Dryman et al., 2016). Theoretical approaches to social anxiety focus on the fear of social evaluation as well as individuals’ self-perceptions and negative evaluation expectations (Clark, 2005; Öztürk, 2014). Identifying the factors associated with higher levels of social anxiety may contribute to a more comprehensive understanding and definition of this phenomenon. Uncovering these factors can provide an important resource for the development of protective and intervention strategies to prevent or reduce social anxiety (Hunger-Schoppe et al., 2022; Wong & Rapee, 2016). When previous studies are examined, intolerance of uncertainty stands out as one of the variables that is associated with higher levels of social anxiety in emerging adults (Saraff et al., 2023). Indeed, according to the etiological explanation model of anxiety, intolerance of uncertainty is a specific factor affecting anxiety (Taylor & Wald, 2003). In this context, individuals with low tolerance for uncertainty tend to report more intense anxiety in uncertain situations, which is also associated with higher levels of social anxiety (Boelen & Reijntjes, 2009; Counsell et al., 2017).

Intolerance of uncertainty is defined by individuals’ intolerance towards uncertain situations and their tendency to perceive such situations as threatening (Buhr & Dugas, 2002). This concept is recognized as an effective factor in the development of anxiety disorders and causes individuals to experience excessive anxiety and stress in the face of uncertain situations (Rifkin & Kendall, 2020; Taylor & Wald, 2003; Uzun, 2024). Theoretically, intolerance of uncertainty is considered a trait that challenges individuals’ resilience to the unknown and negatively affects their risk perception (Buhr & Dugas, 2006; Jensen et al., 2014; Uzun & Arslan, 2025). In this context, it is thought that there may be a significant relationship between intolerance of uncertainty and social anxiety, which is a common problem in emerging adulthood, because low tolerance for uncertainty may lead individuals to feel more anxiety in social situations (Boelen & Reijntjes, 2009; Counsell et al., 2017; Saraff et al., 2023). Although intolerance of uncertainty has been found to be associated with higher levels of social anxiety, there is a need to examine potential mediating variables to better understand this relationship (Bijsterbosch et al., 2020; Boelen et al., 2010; Lowe & Harris, 2019). In particular, it is thought that ruminative thoughts may mediate this relationship between intolerance of uncertainty and social anxiety (Li et al., 2020; Liao & Wei, 2011; Yook et al., 2010). Under conditions of uncertainty, individuals who have negative thoughts about possible social situations tend to report higher levels of social anxiety.

Rumination is defined as a person’s tendency to constantly think about past events, mistakes, or uncertainties about the future and is often associated with negative emotional reactions (Nolen-Hoeksema, 2000). The Response Styles Theory explains why some individuals are more prone to develop psychological problems with ruminative personality traits (Nolen-Hoeksema, 1987) and suggests that distress and stress persist as a result of the individual’s continued focus on negative thoughts (Arslan et al., 2022; Lyubomirsky & Nolen-Hoeksema, 1995). In emerging adults, ruminative beliefs have an important role in the transition to adulthood as a developmental period due to factors such as uncertainty and search for identity, and it has been observed that individuals are more stuck in negative thoughts during this period (Beyers & Luyckx, 2016; Boardman, 2017; Uzun & Arslan, 2024). Moreover, rumination has been found to be positively correlated with emerging adults’ intolerance of uncertainty (Marín-Chollom & Panjwani, 2023); it has been shown that individuals tend to engage in ruminative thinking in the face of uncertain situations (Huang et al., 2019). Similarly, it is known that rumination is positively associated with social anxiety; individuals who engage in more ruminative thinking also tend to report higher levels of anxiety and worry in social settings (Bean & Ciesla, 2024). The possible mediating role of rumination between intolerance of uncertainty and social anxiety is considered a mechanism through which it contributes to individuals’ increased anxiety towards social situations in the face of uncertainty (Li et al., 2020; Liao & Wei, 2011), suggesting that ruminative thoughts may reinforce social anxiety through a lack of tolerance for uncertainty.

### The Present Study

This study aims to fill an important gap in the literature on emerging adults by examining the effect of intolerance of uncertainty on social anxiety and the mediating role of ruminative thoughts in this interaction. When the literature is examined, it is seen that intolerance of uncertainty stands out as a determinant risk factor of social anxiety (Carleton et al., 2010; Saraff et al., 2023); individuals’ intolerance of uncertain situations pushes them to experience more anxiety in social interactions (Boelen & Reijntjes, 2009; Counsell et al., 2017; Whiting et al., 2014). However, the fact that the cognitive processes explaining this effect have not been sufficiently investigated suggests that this important relationship between intolerance of uncertainty and social anxiety should be examined in more detail. In this context, rumination gains theoretical significance as a cognitive process, as individuals who are intolerant of uncertainty tend to focus on their negative thoughts about social situations, which is associated with higher levels of social anxiety (Li et al., 2020; Liao & Wei, 2011; Yook et al., 2010).

Albert Ellis’ Rational-Emotive Behavior Therapy (REBT) and Aaron Beck’s Cognitive-Behavioral Therapy (CBT) are two important theoretical frameworks that emphasize the impact of individuals’ irrational thought patterns and cognitive distortions on emotional well-being (Ellis, 1999; Hollon & Beck, 1994). Intolerance of uncertainty, which is addressed in this study, can be considered a reflection of negative automatic thoughts that individuals develop against uncertainty in the context of REBT and CBT (Robichaud & Dugas, 2006). Likewise, rumination is one of the dysfunctional thought patterns that cognitive therapies often focus on (Watkins, 2016) and is seen as a basic mechanism that maintains emotional problems such as social anxiety (Bean & Ciesla, 2024). Therefore, the present study’s identification of a possible mediating role of rumination in the relationship between intolerance of uncertainty and social anxiety may suggest that REBT and CBT frameworks could be relevant for addressing these cognitive processes through restructuring techniques aimed at improving emotional well-being. Possible findings may indicate that approaches such as REBT and CBT may provide an important basis for developing interventions targeting intolerance of uncertainty and rumination processes.

Conducting this study on emerging adults in Türkiye is important because this age group faces unique developmental and cultural challenges (Atak & Çok, 2010; Doğan & Cebioğlu, 2011). Emerging adulthood is a sensitive period in which individuals experience processes such as gaining identity, developing independence, and making future plans; uncertainty and social pressures in these processes can make social anxiety more prevalent and severe (Arnett, 2000, 2007). This study on emerging adults in Türkiye, which has a collectivist culture, aims to understand how social anxiety tendencies are shaped by cultural and social dynamics specific to Türkiye.

This study contributes to the growing body of literature by investigating the mediating role of rumination in the relationship between intolerance of uncertainty and social anxiety within a Turkish sample of emerging adults. Although previous studies have explored this mechanism in Western and East Asian contexts (e.g., Li et al., 2020; Liao & Wei, 2011), no prior empirical research has, to our knowledge, addressed this mediation model in a Turkish context characterized by high collectivism and low tolerance for uncertainty. Exploring this model in a non-Western, collectivist society adds cultural depth to our understanding of how cognitive vulnerabilities operate across different sociocultural settings. Additionally, by drawing from the theoretical perspectives of REBT and CBT, this study frames intolerance of uncertainty and rumination as maladaptive cognitive processes that may serve as potential targets for therapeutic interventions.

Examining cognitive processes that trigger social anxiety, such as intolerance of uncertainty and ruminative thoughts (Bean & Ciesla, 2024; Saraff et al., 2023), may not only explain social anxiety but also provide important guidance for the development of interventions that increase psychological well-being and resilience to support the healthy social development of emerging adults (Arslan & Wong, 2024). Possible results may draw attention to the necessity of addressing cognitive processes such as intolerance of uncertainty and rumination in intervention programs aiming to reduce social anxiety. This study both fills a theoretical gap in the literature and may provide a new perspective for practitioners (psychologists and counselors) to guide them in managing social anxiety, especially in emerging adults. In light of all of these arguments, the main aim of this research is to investigate how rumination influences the relationship between emerging adults’ social anxiety and their intolerance of uncertainty. In this context, the following hypotheses were developed by considering the recommendations made by Baron and Kenny (1986) for the testing of mediated structural models:

**H_1_:**  *Emerging adults’ intolerance of uncertainty significantly predicts their social anxiety in a positive direction.*

**H_2_:**  *Rumination mediates the relationship between emerging adults’ intolerance of uncertainty and social anxiety.*

## 2. Method

### 2.1. Participants

The study sample consists of 405 emerging adults residing in Türkiye. Schumacker and Lomax (2016) have noted that, for quantitative research involving model building, a sample size between 250 and 500 observations is sufficient. A non-random sampling method, convenience sampling, was used to form the study group. Convenience sampling allows researchers to assemble a sample group by selecting participants who are easiest to reach until the required sample size for the study is achieved (Büyüköztürk et al., 2016). In this sample, 70.60% of the participants (*n* = 286) are female, and 29.40% (*n* = 119) are male. The participants’ ages range from 18 to 25 years, with a mean age of 21.73 (*SD* = 1.87). In terms of socioeconomic status (SES), 8.70% (*n* = 35) reported as poor, 66.20% (*n* = 268) as moderate, and 25.20% (*n* = 102) as good. Additionally, 12.10% of the participants (*n* = 49) reported having a chronic health condition, while 87.90% (*n* = 356) reported no such condition. Furthermore, 29.60% of participants (*n* = 120) indicated that they had previously received psychological support, whereas 70.40% (*n* = 285) reported no history of receiving psychological help.

### 2.2. Measures

*Demographic Information Form:* The researchers created a demographic information form to collect data on the personal traits of the emerging adults in the study group. This form asked about age, gender, socioeconomic situation, history of obtaining psychological support, and chronic health concerns.

*Social Anxiety Scale—Short Form (SAS-SF):* The original 18-item scale developed by La Greca and Lopez (1998) for children was revised by La Greca et al. (2015) to suit secondary school students and adults. Later, Nunes et al. (2018) revised the social anxiety scale further, creating a 12-item short form. The scale was adapted for Turkish culture by Can and Bozgün (2021). The SAS-SF consists of 12 items scored on a 5-point Likert scale. It has three subscales: fear of negative evaluation, social fear and discomfort in new situations, and social fear and discomfort in general situations. Additionally, a total social anxiety score can be obtained from the scale. In the present study, only the total social anxiety score was used rather than the subscales. Responses range from “[1] Strongly disagree” to “[5] Strongly agree”, with possible scores ranging from 12 to 60. Higher scores indicate a higher level of social anxiety. The scale explains 72.27% of the total variance and has an internal consistency coefficient of 0.90 (Can & Bozgün, 2021). To assess the suitability of the SAS-SF for this study, confirmatory factor analysis (CFA) was conducted using maximum likelihood estimation (MLE) in AMOS 24 to verify construct validity. A single-factor model, consistent with the use of the total score, was tested. Results indicated that the values obtained for the SAS-SF met accepted standards of construct validity (χ^2^_[*n*=405]_ = 112.94, *df* = 41, *p* = 0.000, *χ*^2^*/df* = 2.75, RMSEA = 0.07, SRMR = 0.05, CFI = 0.98) and reliability (overall scale *α* = 0.93) as recommended in the literature (Büyüköztürk, 2014; Kline, 2015), supporting its use in this study. Sample items from the scale for further clarity include “[Item-4] I worry about what others think of me” and “[Item-11] I tend to be reserved when in a group of people”.

*Intolerance of Uncertainty Scale (IUS-12):* Developed by Carleton et al. (2007) to assess individuals’ tendencies to exhibit negative emotional, cognitive, and behavioral reactions to uncertain situations, the IUS-12 was adapted into Turkish by Sarıçam et al. (2014). The scale consists of 12 items rated on a 5-point Likert scale, ranging from “[1] Not at all characteristic of me” to “[5] Entirely characteristic of me”. The IUS-12 includes two subscales: prospective anxiety and inhibitory anxiety. A total intolerance of uncertainty score can also be derived from the scale, with possible scores ranging from 12 to 60. Higher scores indicate a higher level of intolerance of uncertainty. The scale explains 78.57% of the total variance and has a general internal consistency coefficient of 0.88 (Sarıçam et al., 2014). To assess the suitability of the IUS-12 for this study, CFA was conducted using MLE in AMOS 24 to verify construct validity. A single-factor model, consistent with the use of the total score, was tested. Results indicated that the values obtained for the IUS-12 met accepted standards of construct validity (χ^2^_[*n*=405]_ = 157.54, *df* = 49, *p* = 0.000, *χ*^2^/*df* = 3.21, RMSEA = 0.07, SRMR = 0.06, CFI = 0.95) and reliability (overall scale *α* = 0.90) as recommended in the literature (Büyüköztürk, 2014; Kline, 2015), supporting its use in this study. Sample items from the scale include “[Item-2] It frustrates me not having all the information I need in a situation” and “[Item-9] When it is time to act, uncertainty paralyzes me”.

*Ruminative Response Scale—Short Form (RRS-SF):* Originally developed by Nolen-Hoeksema and Morrow (1991) to measure individuals’ levels of rumination, the Ruminative Response Scale (22 items) was later revised by Treynor et al. (2003) into a 10-item short form by removing redundant items. The Turkish adaptation of the scale was conducted by Erdur-Baker and Bugay (2012). The RRS-SF consists of 10 items rated on a 4-point Likert scale, with response options ranging from “[1] Never” to “[4] Always”. The scale includes two subscales: brooding and reflective pondering. It can be scored separately by subscale or as a total rumination score. Possible scores range from 10 to 40, with higher scores indicating higher levels of ruminative thoughts. The internal consistency coefficient of the RRS-SF is 0.85 (Erdur-Baker & Bugay, 2012). To assess the suitability of the RRS-SF for this study, CFA was conducted using MLE in AMOS 24 to verify construct validity. A single-factor model, consistent with the use of the total score, was tested. Results showed that the values obtained for the RRS-SF met accepted standards of construct validity (*χ*^2^_[*n*=405]_ = 104.92, *df* = 31, *p* = 0.000, *χ*^2^/*df* = 3.38, RMSEA = 0.07, SRMR = 0.03, CFI = 0.95) and reliability (overall scale *α* = 0.87) as outlined in the literature (Büyüköztürk, 2014; Kline, 2015), supporting its use in this study. Sample items from the RRS-SF include “[Item-1] How often do you think ‘What did I do to deserve this?’” and “[Item-8] How often do you think ‘Why can’t I handle things better?’”.

### 2.3. Data Collection

Data were collected through an online survey created using Google Forms. The target group consisted of emerging adults aged between 18 and 25 years residing in Türkiye. This age range was selected in line with the developmental stage of emerging adulthood, which is characterized by significant identity exploration, increased emotional vulnerability, and intensified social experiences (Arnett, 2000, 2007). This period is considered particularly relevant for studying constructs such as intolerance of uncertainty, rumination, and social anxiety. Participants were not limited to university students; the sample included individuals who were currently enrolled in university, recent graduates, unemployed individuals, and those working in various sectors.

The recruitment process was conducted through multiple digital channels. The survey link was disseminated via email and shared across popular social media platforms, including Facebook and WhatsApp. In particular, the link was posted in relevant youth-focused groups and pages, such as university alumni networks, student associations, and job-seeking or peer-support communities. Prior to posting, permission was obtained from the administrators or moderators of these online groups, where applicable.

The recruitment messages clearly stated the voluntary nature of participation, the inclusion criterion of being between 18 and 25 years of age, and included a link to the online informed consent form. Participants who agreed to take part accessed the survey by clicking the provided link, read the consent information, and indicated their approval before proceeding to the questionnaire. Anonymity and confidentiality were assured throughout the process, and no personal identifiers were collected. No incentives were offered for participation. In addition to the age requirement, participants were also required to self-report that they did not have any serious psychological disorders, were not currently undergoing psychiatric treatment, and were not using psychiatric medication that could potentially affect their cognitive processing or ability to complete the questionnaire. These screening questions were presented at the beginning of the survey to ensure that only eligible individuals participated.

As part of the inclusion and exclusion criteria, only participants between the ages of 18 and 25 and residing in Türkiye were eligible to participate. Individuals outside the specified age range, those who did not provide informed consent, or those who would report serious psychological conditions or psychiatric treatment/medication use were to be excluded. However, no participants reported any serious psychological disorders or psychiatric treatment/medication use during the self-report screening process. The only exclusion occurred based on the age criterion: Specifically, 12 participants who reported being between 26 and 32 years old were identified and excluded from the dataset prior to analysis, even though they had completed the questionnaire in full. Furthermore, the sociodemographic characteristics of the sample, including gender, socioeconomic status, history of psychological support, and chronic health conditions, are reported in detail in the Participants Section to aid in evaluating the generalizability of the findings.

All procedures involving human participants were carried out in accordance with the 1964 Declaration of Helsinki and its later amendments, as well as the ethical standards of the institutional and/or national research committee. Ethical approval for this study was obtained on December 5, 2024, from the Ethics Committee of the author’s institution (reference number: 2024-217). Following the approval, data collection was conducted between December 2024 and February 2025. During this period, data were collected, analyzed, and this study was written up for reporting.

### 2.4. Data Analyses

Preliminary analyses were carried out to test normality, analyze descriptive statistics, and look at the correlation coefficients between the study variables before looking at the suggested hypothetical mediation model. Skewness and kurtosis measurements that met the predetermined threshold values were used to determine normality (Kline, 2015). The associations between the research variables were then examined using Pearson product-moment correlation analysis. To test the hypothesized mediation model examining the mediating role of rumination in the relationship between intolerance of uncertainty and social anxiety, PROCESS macro (Model 4) for SPSS version 3.4 was used (Hayes, 2018). Model results were interpreted based on standardized path estimates (*β*) and squared multiple correlations (*R*^2^). According to Cohen (1988), *R*^2^ is categorized into small (0.01–0.06), medium (0.06–0.14), and large (≥0.14) effect sizes. Additionally, the bootstrap method with 5000 resamples was used to calculate 95% confidence intervals (CIs) for the indirect effect (Hayes, 2018). Another parameter used in evaluating the mediation models is the completely standardized effect size (*K*^2^ [Kappa]); values of *K*^2^ close to 0.01 indicate a small effect, 0.09 a medium effect, and 0.25 a large effect (Preacher & Kelley, 2011). All statistical analyses were conducted using SPSS version 25.

## 3. Results

### 3.1. Preliminary Analyses

Preliminary analysis results indicated that kurtosis values ranged from −0.59 to −0.32, and skewness values ranged from −0.24 to 0.33, suggesting a normal distribution for all variables. These findings are consistent with Kline’s (2015) recommendation that skewness values should be below |3| and kurtosis values below |10|, supporting the assumption of normality. The internal reliability estimates for the scales, presented in Table 1, demonstrated strong reliability. Additionally, Pearson correlation analysis revealed significant positive relationships with medium effect sizes between social anxiety and intolerance of uncertainty (*r* = 0.39, *p* < 0.001) and rumination (*r* = 0.48, *p* < 0.001) (see Table 1). Furthermore, rumination showed a significant positive correlation with intolerance of uncertainty (*r* = 0.53, *p* < 0.001), indicating a medium effect size.

### 3.2. Testing the Proposed Model

After examining the preliminary analyses, we investigated whether intolerance of uncertainty is associated with social anxiety in emerging adults through rumination, as detailed in Table 2. Hypothetical mediation analyses revealed that intolerance of uncertainty significantly and positively predicted rumination (*β* = 0.36, *p* < 0.001), explaining 30% of the variance in rumination. Additionally, intolerance of uncertainty emerged as a significant positive predictor of social anxiety (*β* = 0.22, *p* < 0.001). Furthermore, rumination was found to be a positive predictor of social anxiety (*β* = 0.63, *p* < 0.001). The combined effect of intolerance of uncertainty and rumination explained 26% of the variance in social anxiety among emerging adults.

Table 3 presents the unstandardized total, direct, and indirect effects predicting social anxiety scores, along with 95% bias-corrected confidence intervals. The findings highlight that intolerance of uncertainty is directly associated with social anxiety (*β* = 0.22, *p* < 0.001, *R*^2^ = 0.17), supporting *H*_1_. Additionally, intolerance of uncertainty is indirectly associated with social anxiety through rumination (*β* = 0.23, *p* < 0.001), indicating that rumination serves as a hypothesized mediator of the effect of intolerance of uncertainty on social anxiety in emerging adults. Therefore, *H*_2_ is also supported.

**Figure 1:**
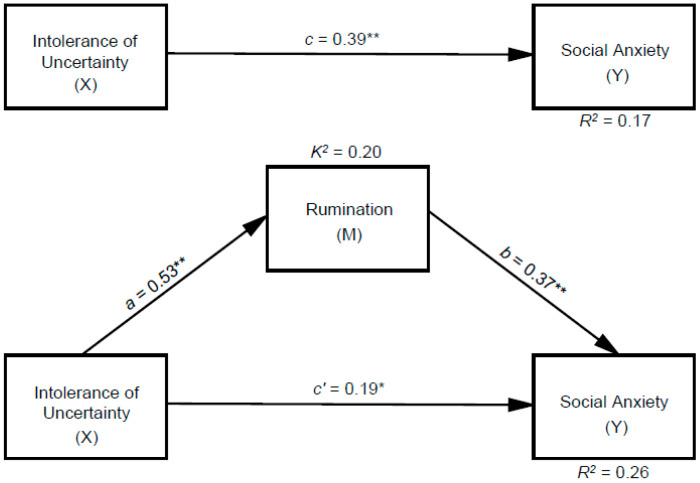
Hypothetical mediation model indicating the standardized associations between variables. ** *p* < 0.001, * *p* < 0.05.

As shown by the standardized coefficients in Figure 1, the inclusion of rumination in the model reduced the path coefficient from intolerance of uncertainty to social anxiety from 0.39 to 0.19 while maintaining statistical significance. In this context, it can be concluded that rumination plays a partial mediating role in the relationship between intolerance of uncertainty and social anxiety.

An additional parameter to consider when evaluating the hypothetical mediation model is the completely standardized effect size (*K*^2^). Table 2 presents the values related to effect size for the significant mediation observed between intolerance of uncertainty and social anxiety through the hypothesized mediator, rumination. An examination of Table 2 indicates that the effect of rumination in the proposed mediation model is large (*K*^2^ = 0.20).

### 3.3. Supplementary Analysis: Controlling for Gender, Psychological Support, and SES

To further test the robustness of the proposed mediation model, an additional analysis was conducted in which gender, psychological support history, and SES were included as covariates. The results of this supplementary analysis are presented in Table 2.

Intolerance of uncertainty remained a significant predictor of rumination (*β* = 0.36, *p* < 0.001). While psychological support history negatively predicted rumination (*β* = −1.39, *p* = 0.019), the effects of gender (*β* = −1.03, *p* = 0.083) and SES (*β* = −0.22, *p* = 0.598) on rumination were not significant.

In the model predicting social anxiety, the key pathways remained stable and statistically significant. Intolerance of uncertainty continued to directly predict social anxiety (*β* = 0.22, *p* < 0.001), and rumination was also a significant predictor (*β* = 0.63, *p* < 0.001). Among the covariates, neither gender (*β* = −0.14, *p* = 0.894) nor psychological support history (*β* = −0.39, *p* = 0.706) significantly predicted social anxiety. SES had a marginal negative association with social anxiety (*β* = −1.40, *p* = 0.062).

Finally, the indirect effect of intolerance of uncertainty on social anxiety through rumination remained statistically significant and substantial after controlling for all covariates (*β* = 0.20, 95% CI [0.14 to 0.26]). These findings indicate that the mediation model is robust, and the primary pathways remain valid even when potential confounding variables are taken into account. Thus, the hypothesized role of rumination as a partial mediator between intolerance of uncertainty and social anxiety was supported across all analyses.

## 4. Discussion

Understanding the cognitive processes associated with higher levels of social anxiety in emerging adulthood is important for informing interventions that aim to support psychological well-being and resilience. Accordingly, our study investigated how social anxiety levels of individuals who are intolerant of uncertainty are affected by ruminative thoughts. The results of this study indicate that intolerance of uncertainty is associated with higher levels of social anxiety in emerging adults and that ruminative thoughts may play a potential mediating role in this relationship. In this context, the results indicate that the effects of intolerance of uncertainty and ruminative thoughts on social anxiety should be better understood.

The first important result obtained in this study is that the level of intolerance of uncertainty in emerging adults is positively related to social anxiety, and intolerance of uncertainty significantly predicts social anxiety. This result suggests that individuals with low tolerance for uncertainty tend to report higher levels of anxiety in social interactions. Individuals with high sensitivity to uncertainty often perceive social environments as more threatening, which is associated with elevated levels of social anxiety. There are also some studies in the literature that reach results consistent with the findings of this study. For example, Saraff et al. (2023) conducted an experimental study with university students with clinical social anxiety symptoms. In the study, participants were randomly assigned to two conditions in which the level of uncertainty was manipulated and how social anxiety was affected in these conditions was examined. In the high uncertainty condition, intolerance of uncertainty significantly predicted social anxiety. Similarly, Boelen and Reijntjes (2009) found that intolerance of uncertainty significantly predicted social anxiety symptoms. It has been shown that participants’ sensitivity to uncertainty is related to social anxiety and that this sensitivity may increase the severity of social anxiety. In addition, the results obtained from Counsell et al.’s (2017) study indicate that individuals with low tolerance for uncertainty experience social anxiety symptoms more intensely.

The fact that the current study was conducted in Türkiye draws attention to the social norms of Turkish culture and the factors that trigger social anxiety. Values such as social acceptance, giving importance to the opinions of others, and avoiding exclusion are quite common in Turkish culture (Gerçik, 2020). These social norms may cause individuals who do not know what they will encounter in the social environment that they will enter to feel inadequate in social environments and fear being evaluated negatively, which may increase their social anxiety levels. In this context, in a culture with collectivist values like Türkiye, intolerance of uncertainty stands out as an important factor that is associated with higher levels of social anxiety among individuals.

The second important result obtained in this study suggests that rumination may serve as a significant mediator in the association between intolerance of uncertainty and social anxiety. This result suggests that individuals with low tolerance for uncertainty tend to report higher levels of social anxiety, particularly when they focus on negative thoughts related to social situations. In other words, intolerance of uncertainty may be associated with elevated social anxiety, potentially through its relationship with increased ruminative thinking, which could contribute to a more complex cognitive–emotional experience. This result suggests that social anxiety is not only a matter of coping with an external situation but also that internal cognitive processes are effective in this relationship. Similar to the results of this study, there are some findings in the literature that rumination may mediate the relationship between intolerance of uncertainty and social anxiety. In a study conducted on university students in China, Li et al. (2020) found that rumination played a partial mediating role in the relationship between intolerance of uncertainty and social anxiety. In a similar study conducted with university students, Liao and Wei (2011) concluded that high levels of rumination strengthened the relationship between intolerance of uncertainty and anxiety and that rumination partially mediated the effect of intolerance of uncertainty on anxiety. These results suggest that intolerance of uncertainty may be associated with higher levels of anxiety symptoms, potentially through its relationship with negative cognitive processes.

The fact that the current study was conducted in Türkiye is important in terms of evaluating the results in a cultural context. The fact that Turkish culture tends to have a low tolerance for uncertainty (Hofstede, 1980) suggests that this intolerance may increase individuals’ anxiety and ruminative thinking tendencies towards social situations. Therefore, this study makes an important contribution to understanding the social anxiety dynamics of emerging adults in Türkiye; at the same time, it draws attention to the importance of cultural sensitivity in interventions to reduce social anxiety.

Although our findings are consistent with earlier research, this study presents several unique contributions. First, it is the first to empirically test this mediation model in a Turkish sample of emerging adults, a population that faces developmental and cultural stressors specific to collectivist societies. Cultural values such as social approval, the fear of exclusion, and low tolerance for uncertainty may intensify the effects of maladaptive cognitions like rumination and intolerance of uncertainty. Second, this study offers a theoretically integrated perspective by applying REBT and CBT frameworks to interpret how these cognitive patterns contribute to social anxiety. Together, these cultural and theoretical dimensions provide a more comprehensive understanding of the mechanisms that underlie social anxiety and offer direction for culturally sensitive interventions.

### 4.1. Limitations and Implications

Although this study found some significant outcomes, it contains a number of methodological flaws. First, a cross-sectional technique was used to perform the proposed model, which limits the ability to infer causality between the variables under examination, even though a mediation model was tested. Using longitudinal designs, more research is needed to shed more light on the connections between the studied variables. Another limitation of this study is that the data used in the study were collected using self-report scales. Although these instruments demonstrated good psychometric properties within Turkish samples—including confirmatory factor analysis and internal consistency estimates—they have not been validated through clinical observations or structured interviews. Therefore, potential biases such as social desirability and recall inaccuracies may have influenced the participants’ responses. It was assumed that the individuals participating in this study responded truthfully and sincerely to the measurement tools used in this study.

In addition, the study group of this research was limited to individuals living in Türkiye and in emerging adulthood. Emerging adults from different cultures and geographies were not included in this study. In this context, it can be said that the generalizability of the results of this study is limited to similar collectivist cultures. Given the influence of cultural factors on individuals’ emotions, thoughts, and behaviors, it is recommended that future research systematically examine these findings in different cultural contexts, including both collectivist and individualist societies. In addition, the convenience sampling method also hinders the generalizability of the study findings. The use of a non-random, convenience sampling method also raises concerns about potential selection biases, and the demographic composition of the sample limits its representativeness. To increase the external validity of the findings, it is important to replicate the mediation effect using larger and more diverse samples of adults. In addition, it is imperative to validate the study results across developmental stages from adolescence to later adulthood before extrapolating the findings.

Furthermore, while intolerance of uncertainty and rumination together accounted for 26% of the variance in social anxiety, a substantial proportion (74%) of the variance remains unexplained. This indicates that other psychological, environmental, or interpersonal variables—such as self-esteem, attachment styles, perfectionism, or past trauma—may also contribute significantly to social anxiety in emerging adults. This unexplained variance should be acknowledged as a key limitation, and future studies are encouraged to explore additional predictors to build a more comprehensive explanatory model of social anxiety.

The last limitation concerns the use of only total scores for the scales employed (SAS-SF, RRS-SF, and IUS-12), without incorporating their subscales. This decision was made to maintain the conceptual clarity and parsimony of the mediation model. However, it is acknowledged that analyzing subscales could have offered more detailed insights into the specific mechanisms underlying the observed relationships. Therefore, future studies are encouraged to examine the roles of individual subdimensions to enhance the explanatory power of such models. Based on the results and limitations of the current study, some suggestions have been developed both for new researchers who plan to conduct studies on this topic and for practitioners in the field.

The results of this study highlight the potential relevance of addressing intolerance of uncertainty and ruminative thoughts within interventions aimed at supporting individuals experiencing elevated social anxiety. Accordingly, intervention programs aimed at reducing intolerance of uncertainty and managing ruminative thoughts can be designed to help emerging adults cope with their social anxiety and support their psychological well-being. Rational-Emotive and Cognitive-Behavioral techniques can be used in the implementation of these intervention programs. Psychologists and counselors can integrate techniques to develop coping skills for intolerance of uncertainty and ruminative thoughts into therapy processes addressing social anxiety. These skills can help individuals manage social anxiety more effectively. Group work that addresses intolerance of uncertainty and rumination processes may aim to benefit from interaction and peer support in the group environment in coping with social anxiety. Thus, participants can be provided with an empowered support environment in managing their social anxiety. The results obtained in this study indicate that the cognitive mechanisms affecting social anxiety need to be better understood. In this regard, future studies should not only continue to explore cognitive mediators such as rumination but also test broader psychological frameworks that include variables like emotion regulation, self-concept clarity, or interpersonal sensitivity. In particular, psychological variables such as self-esteem, depressive symptoms, or perceived social support may either mediate or moderate these relationships. Including these factors in future models could offer a more nuanced understanding of how intolerance of uncertainty and rumination contribute to social anxiety among emerging adults.

### 4.2. Conclusions

The current study is thought to provide a deeper understanding of the relationship between social anxiety and intolerance of uncertainty. In this study, intolerance of uncertainty was found to be significantly associated with higher levels of social anxiety among emerging adults, a period marked by intensified social interactions and challenges related to academic or occupational roles. This association also appeared to be linked with cognitive processes such as rumination, which may contribute to the complexity of individuals’ emotional experiences. The findings suggest that individuals with higher intolerance of uncertainty tend to report greater anxiety in social settings, and this pattern appears to be associated with increased ruminative thinking. Especially in collectivist cultures with low tolerance for uncertainty, such as Türkiye (Hofstede, 1980), higher levels of social anxiety may be observed. This study emphasizes the importance of considering cultural factors in this process by addressing the social anxiety dynamics of emerging adults in Türkiye in a cultural context. As a result, it should be noted that focusing only on individuals’ social skills will not be sufficient in interventions to reduce social anxiety; at the same time, it is important to include strategies for managing intolerance of uncertainty and ruminative thoughts in the process.

## Figures and Tables

**Table 1:** Descriptive statistics and correlation coefficients for the variables.

	Descriptive Statistics					Correlation Coefficients (*r*)		
Scales	α	*M*	*SD*	*g*_1_	*g*_2_	1.	2.	3.
1. SA	0.93	31.99	10.90	0.33	−0.47	-	0.39 **	0.48 **
2. IU	0.90	40.49	9.36	−0.25	−0.32		-	0.53 **
3. R	0.87	25.76	6.43	0.05	−0.59			-

*Note. g*_1_, skewness; *g*_2_, kurtosis; SA, social anxiety; IU, intolerance of uncertainty; and R, rumination. ** *p* < 0.001.

**Table 2:** Unstandardized coefficients for the mediation model with covariates.

Antecedent		Consequent					
		Coeff.	*SE*	*t*	*p*	BootLLCI	BootULCI
		*M* (Rumination)					
*X* (Intolerance of Uncertainty)	*a*	0.36	0.03	12.59	<0.001	0.31	0.42
Gender	*-*	−1.03	0.59	−1.74	0.083	−2.20	0.13
SES	*-*	−0.23	0.43	−0.53	0.599	−1.07	0.62
Psychological Support	*-*	−1.39	0.59	−2.36	0.019	−2.55	−0.23
Constant	*İ_M_*	15.51	2.18	7.11	<0.001	11.23	19.80
		*R*^2^ = 0.30					
		*F*_(4–400)_ = 43.01; *p* < 0.001					
		*Y* (Social Anxiety)					
*X* (Intolerance of Uncertainty)	*c’*	0.22	0.06	3.74	<0.001	0.11	0.34
*M* (Rumination)	*b*	0.63	0.09	7.23	<0.001	0.46	0.80
Gender	*-*	−0.14	1.04	−0.13	0.894	−2.18	1.90
SES	*-*	−1.40	0.75	−1.87	0.062	−2.87	0.07
Psychological Support	*-*	−0.39	1.04	−0.38	0.706	−2.43	1.64
Constant	*İ_Y_*	12.08	4.03	2.99	0.003	4.16	20.01
		*R*^2^ = 0.26					
		*F*_(5–399)_ = 28.77; *p* < 0.001					
Completely Standardized Indirect Effect	*ab*	0.20	0.03	-	-	0.14	0.26

*Note. SE* = standard error; Coeff = unstandardized coefficient; *X* = independent variable; *M* = mediator variables; and *Y* = outcome or dependent variable. Covariates included gender, SES, and psychological support history.

**Table 3:** Unstandardized total, direct, and indirect effects for the model.

Path	Effect	*SE*	BootLLCI	BootULCI
Total Effect (IU → SA)	0.45	0.05	0.35	0.56
Direct Effect (IU → SA)	0.22	0.06	0.11	0.34
Indirect Effect (IU → R → SA)	0.23	0.04	0.16	0.31

*Note.* IU = intolerance of uncertainty; SA = social anxiety; and R, rumination. Number of bootstrap samples for percentile bootstrap confidence intervals: 5000.

## Data Availability

Data are available upon reasonable request.
